# Associations between metabolic syndrome and regional brain iron depositions and cognitive function in middle‐aged and older adults: A two multinational cohort study

**DOI:** 10.1002/alz.71080

**Published:** 2026-01-08

**Authors:** Xinyue Zhang, Linfeng Yang, Na Wang, Yuanyuan Wang, Qihao Zhang, Zhenyu Cheng, Yiwen Chen, Pengcheng Liang, Meng Li, Lingfei Guo, Changhu Liang

**Affiliations:** ^1^ Key Laboratory of Endocrine Glucose & Lipids Metabolism and Brain Aging Department of Radiology Ministry of Education Shandong Provincial Hospital Affiliated to Shandong First Medical University Jinan Shandong China; ^2^ Department of Radiology National Center of Gerontology Institute of Geriatric Medicine Beijing Hospital Chinese Academy of Medical Sciences & Peking Union Medical College Beijing China; ^3^ Department of Radiology Jinan Maternity and Child Care Hospital Affiliated to Shandong First Medical University Jinan Shandong China; ^4^ School of Medical Imaging Binzhou medical university Yantai Shandong China; ^5^ Department of Radiology Weill Cornell Medical College New York USA; ^6^ Department of Psychiatry and Psychotherapy Jena University Hospital Jena Germany; ^7^ Center for Intervention and Research on adaptive and maladaptive brain Circuits underlying mental health (C‐I‐R‐C) Halle‐Jena‐Magdeburg Jena Germany

**Keywords:** brain iron, cognitive function, dual cohort study, metabolic syndrome, voxel‐based analysis

## Abstract

**INTRODUCTION:**

The alterations of regional brain iron in patients with metabolic syndrome (Mets) and its relationship with cognitive function remain unclear.

**METHODS:**

These analyses utilized data from two prospective cohorts, from the UK Biobank (UKB) (21,346 participants) and from Jinan, China (224 participants). We capitalized brain iron in the striatum and thalamus of UKB. Then voxel‐based analysis of quantitative susceptibility mapping (QSM) was used to detect regional susceptibility value alteration in our cohort and their relationship with cognitive function was assessed using linear regression.

**RESULTS:**

Mets patients exhibited iron deposition in the striatum and thalamus, which was mainly associated with hyperglycemia and elevated triglycerides among the risk factors in UKB. Mets patients exhibited iron deposition in the right caudate nucleus, which was associated with cognitive decline in the Jinan cohort.

**DISCUSSION:**

Regional brain iron deposition might serve as a neuroimaging biomarker of Mets severity and a potential mechanism for cognitive decline.

**Highlights:**

- Iron deposition in deep gray matter nuclei was found in patients with metabolic syndrome (Mets) in both UK Biobank (UKB) and Jinan cohort.
- Iron deposition in the deep gray matter nuclei of Mets in UKB was mainly related to hyperglycemia and elevated triglycerides among metabolic risk factors.
- The UKB longitudinal study found that the susceptibility values of the caudate nucleus and putamen of Mets patients increased significantly with age compared to healthy controls.
- Patients with Mets exhibited poorer cognitive function, which was closely associated with iron deposition in the right caudate nucleus in the Jinan cohort.
- The association between iron deposition and cognition was more concentrated in the elderly, smokers and drinkers, and those with low years of education.

## 1 BACKGROUND

Metabolic syndrome (Mets) is a complex disease characterized by a constellation of metabolic disturbances, including elevated triglycerides, low high‐density lipoprotein (HDL) levels, high blood pressure, dysregulated glucose homeostasis and abdominal obesity.^1^, ^2^ The global prevalence of Mets is estimated to account for approximately a quarter of the world's population and is one of the major public health burdens globally.^3^ Mets is frequently considered a “predisease” state that increases the risk of cerebrovascular disease and is related to cognitive decline and neurological disorders such as dementia and stroke.^4^, ^5^ Advanced neuroimaging techniques have recently been used to determine the relationship between Mets and neurological disorders. However, the associations among Mets, brain iron homeostasis, and brain aging remain unclear.

The role of brain iron deposits in metabolic disease risk factors such as obesity, insulin resistance (IR), and neurodegenerative disease pathogenesis has received increasing attention. Iron homeostasis plays a crucial role in maintaining the normal physiological functions of nerve cells, such as DNA, neurotransmitter synthesis, myelin respiration, and oxygen transport.^6^ However, excessive iron accumulation in specific brain regions is associated with homeostatic disruption and cellular damage and often occurs in the pathogenesis of neurodegenerative diseases.^7^ Several studies have shown that iron levels in the caudate nucleus, lenticular nucleus, and hippocampus are significantly increased in obese subjects and are associated with poorer cognitive performance.^8^, ^9^ The results of animal experiments indicated that diabetes mellitus and hypercholesterolemia may impair brain function through oxidative stress and cholinergic dysfunction.^10^ Progressive iron deposition has been linked to the development of type 2 diabetes, which affects the microstructure and function of the brain and occurs mainly in obese people.^11^, ^12^ A previous study indicated that brain iron in the caudate nucleus, putamen and dentate nucleus was significantly elevated in hypertensive patients and was associated with cognitive impairment.^13^ The understanding of the impact of each Mets component on iron homeostasis in deep gray matter nuclei was limited. Moreover, many metabolic abnormalities cooccur,^14^, ^15^ and as a collection of these conditions, Mets has not been studied extensively in large cohorts.

The striatum, which is part of the basal ganglia, is composed of the caudate nuclei and putamen and is responsible for regulating movement, the reward system, involves cognitive functions such as procedural learning and working memory.^16^, ^17^ The thalamus is the main information relay station and is involved in motor control and the cognitive function of the body.^18^ Research has suggested that the severity of Mets is associated with changes in the volume of the basal ganglia, especially the striatum and thalamus.^19^ Specifically, a larger waist circumference was linked to a larger volume of the right caudate nucleus, bilateral putamen, and thalamus. The volumes of the putamen and pallidum are significantly correlated with triglyceride and HDL levels.^20^ High‐field imaging and spectroscopy studies have revealed that changes in blood glucose levels are associated with reduced thalamic activation in patients with type 1 diabetes.^21^ Notably, the regional distribution of total iron in a healthy adult brain is heterogeneous, and the highest iron concentration is detected in the basal ganglia, especially in the striatum.^22^ Nevertheless, few studies have evaluated whether Mets and its five metabolic components affect striatal and thalamic iron homeostasis.

**RESEARCH IN CONTEXT**

1. **Systematic review**: Metabolic syndrome (Mets) is a complex disease characterized by a constellation of metabolic disturbances and frequently considered a “predisease” state that increases the risk of cerebrovascular disease and related to cognitive decline and neurological disorders such as dementia and stroke. According to our literature review, few studies have been conducted simultaneously in Asian and Western cohorts, and explored the manifestation of brain iron deposition in the cognitive decline of patients with Mets.
2. **Interpretation**: We demonstrated that Mets can contribute to iron deposition in the putamen, caudate nucleus and pallidum and thalamus in UK Biobank (UKB) and right caudate nucleus in Jinan cohort. The susceptibility values of the caudate nucleus and putamen of Mets patients increased significantly with age compared to healthy controls in the UKB longitudinal data. We suggested the potential of the deep gray matter nuclei iron deposition as a biomarker for Mets, indicating that iron deposition in the right caudate nucleus was associated with a higher risk of cognitive progression.
3. **Future directions**: The effect on the cortical susceptibility value will continue to be investigated in our study cohort in the future and we will perform precise segmentation of nuclear subregions. It is great significance to promote the effective diagnosis and prognosis of Mets in both Western and Eastern populations.

To explore the above questions further, we first aimed to use UK Biobank (UKB) with a wide range of measures, including blood measures, demographics, socioeconomic status, lifestyle factors, and brain magnetic resonance imaging (MRI) data, to investigate the association of Mets with brain iron deposition in the striatum and thalamus. Subsequently, we aimed to verify the findings in the UKB and further explore its relationship with cognitive function in the Jinan cohort. We hypothesized that regional brain iron deposition occurs in Mets, potentially contributing to cognitive progression.

## 2 METHODS

### 2.1 Participants

**FIGURE 1:**
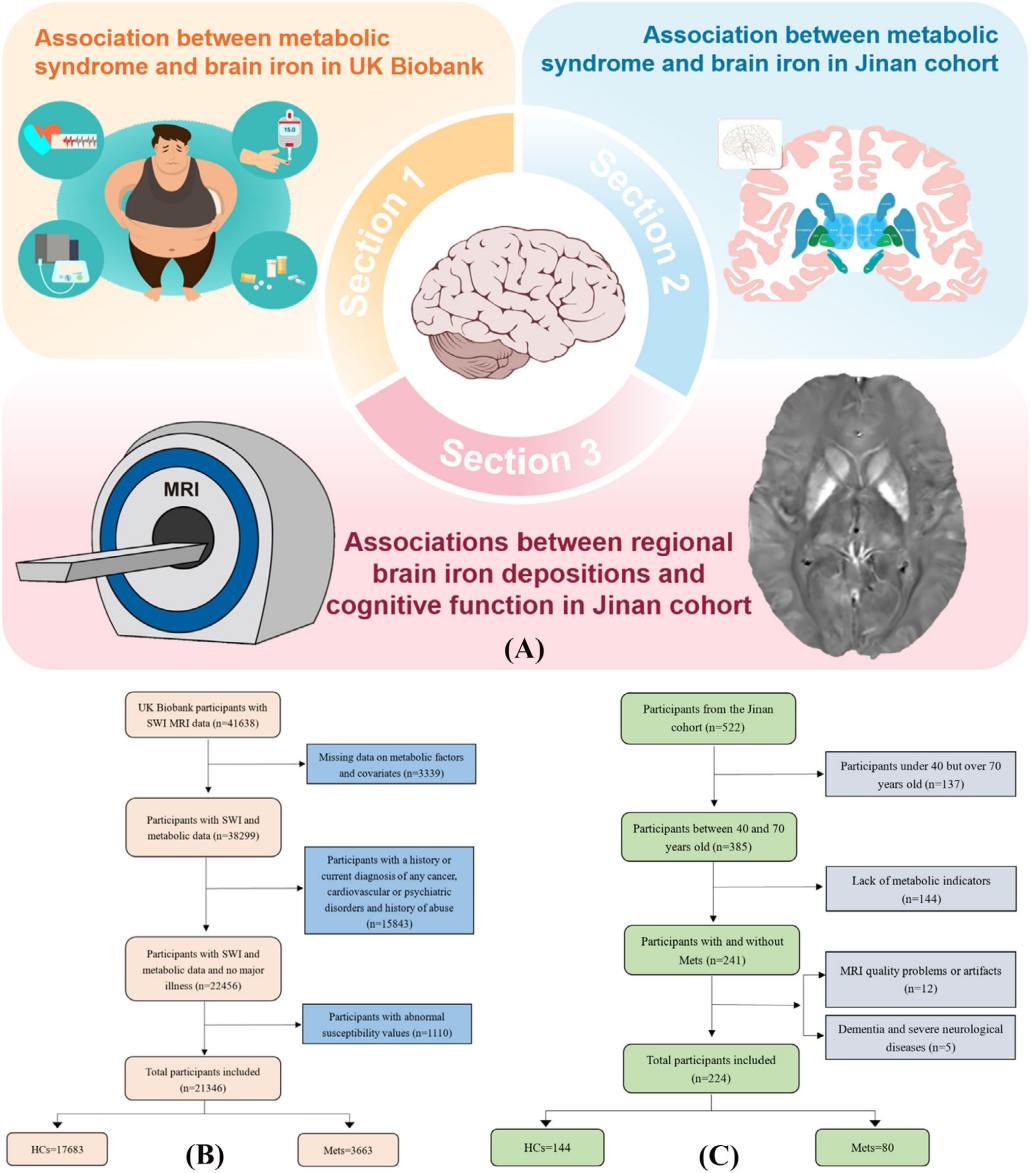
The flowchart of the study design. (A) The study design map. The illustration of the brain was sourced from https://scidraw.io/. The participants selection process of the UK biobank (B) and Jinan cohort (C). HCs, healthy controls; Mets, metabolic syndrome.

This study used data from UKB (https://www.ukbiobank.ac.uk) and the Jinan cohort (ISRCTN13222678), which were prospective cohorts conducted in the United Kingdom and Jinan, China, respectively. Written informed consent was signed by all participants in both cohorts. UKB received ethical approval from the Ethics Approval Committee (ref. 11/NW/0382) and the Jinan cohort was approved by the Ethics Committee of Shandong Provincial Hospital Affiliated to Shandong First Medical University (No.2019‐002). The study designs of both cohorts were described in Figure 1A.

The selection process of the study population was shown in Figure 1B,C. The UKB collected comprehensive biological and medical information on approximately 500,000 participants between 40 and 69 years old. To date, approximately 50,000 participants have undergone brain MRI scans. We included participants with susceptibility‐weighted MRI (SWI) data (*n* = 41638) and removed participants due to data deficiency and various diseases (for details, see Supporting Information). In the Jinan cohort, approximately 522 individuals collected MRI brain imaging data, as well as other health‐related data. We included adults aged 40 to 70 years old (*n* = 385) and excluded participants with incomplete data (*n* = 144) and self‐reported or hospital‐recorded history of dementia or other serious neurological disorders (*n* = 5). Finally, 21,346 (10,126 males) UKB participants and 224 (113 males) participants from the Jinan cohort were included after applying the inclusion/exclusion criteria. Then, we included 861 participants with longitudinal follow‐up data on quantitative susceptibility mapping (QSM) images in the UKB, including the Mets group (294 participants, 175 males, mean follow‐up time of 2.68 years) and the healthy control (HC) group (567 participants,191 males, mean follow‐up time of 2.63 years).

### 2.2 Mets

The participants were categorized into the HCs and Mets groups. Mets of UKB participants was defined by the National Cholesterol Education Program Adult Treatment Panel III (NCEP‐ATP III).^23^ The presence of three or more of the following five components constitutes a Mets diagnosis:(1) elevated fasting blood glucose (≥5.6 mmol/L); (2) reduced HDL (< 1.0 mmol/L in males; < 1.3 mmol/L in females); (3) elevated triglycerides (TG) (≥1.7 mmol/L); (4) elevated blood pressure (≥130 mmHg systolic blood pressure and/or ≥85 mmHg diastolic blood pressure) or antihypertensive medication use; and (5) abdominal obesity (elevated waist circumference: ≥102 cm in males and ≥88 cm in females). The measurement details were given in the Supporting Information.

Mets of the participants in the Jinan cohort was defined based on the diagnostic criteria proposed by the Diabetes Society of the Chinese Medical Association.^24^ The presence of three or more of the following five components was included in the Mets group:(1) elevated fasting blood glucose ≥6.1 mmol/L or (and) those who have been diagnosed with diabetes and are receiving treatment; (2) fasting blood TG≥1.7 mmol/L or (and) fasting blood HDL < 0.9 mmol/L (male) or < 1.0 mmol/L (female); (3) systolic blood pressure≥140 mmHg or diastolic blood pressure≥90 mmHg, or those who have been diagnosed with hypertension and are receiving treatment; (4) body mass index (BMI) ≥ 25 kg/m^2^.

### 2.3 Cognitive assessment

In the Jinan cohort, the Beijing version of Montreal Cognitive Assessment (MoCA) (www.mocatest.org) was used to assess global cognitive function of participants.^25^ Additionally, the cognitive sub‐assessments included the Symbol Digit Modalities Test (SDMT), Trail Making Test (TMT), Stroop Color‐Word Test (SCWT) and the Rey Auditory Verbal Learning Test (AVLT) to assess executive function, memory, visual search, information processing speed, and attention.^26^, ^27^, ^28^, ^29^ We performed cognitive tests and scored according to international standards, as detailed in the Supporting Information.

### 2.4 MRI acquisition and processing

The participants underwent brain MRI at three imaging centers (Newcastle‐upon‐Tyne, Stockport, or Reading) via the same Siemens Skyra 3T scanner with a standard 32‐channel head coil in UKB. This study used SWI data (3D gradient recalled echo [3D‐GRE], voxel size = 0.8*0.8*3.0 mm, matrix size  =  256 * 288 * 48, TE1/TE2/TR = 9.4/20/27 ms) for sensitivity measurements of magnetic tissue components.^30^ The QSM used for brain iron content determination were derived from SWI data, which are influenced mainly by myelin storage proteins and iron in deoxyhemoglobin. QSM relies on phase images acquired from individual coil channels, which are merged, masked, and unwrapped. Susceptibility values are calculated through the QSM process, which involves background field removal, dipole inversion, and cerebrospinal fluid (CSF) referencing,^31^ as detailed in the Supporting Information. Based on our research objective, we extracted the susceptibility values and corresponding volumes of the bilateral Cau, putamen, pallidum, and thalamus, as well as normalized gray matter volume from the UKB, for the cross‐sectional and longitudinal analyses. The susceptibility values of the other gray matter brain regions: accumbens, amygdala, hippocampus, and substantia nigra in the UKB were extracted for supplementary analysis. Given the strong correlation between the left and right hemisphere iron deposition measurements (correlations 0.48 to 0.79, both *p* < 0.001), the means of the left and right hemisphere‐matched variables were used in the analyses.

Brain scans were also performed using a Siemens Skyra 3.0T scanner with a 32‐channel head coil in the Jinan cohort. QSM was performed using a 3D multi‐echo gradient echo (mGRE) sequence (TR = 50 ms, TE = 6.8 ms, echo interval = 4.1 ms, 10 echoes, flip angle = 15°, voxel size = 1 × 1 × 2 mm^3^). Anatomical structure imaging was obtained by a 3D T1‐weighted magnetization‐prepared rapid gradient echo sequence. We simultaneously scanned T2‐weighted (T2W) turbine spin echo, T2W fluid‐attenuated inversion recovery, and diffusion‐weighted imaging sequences to search for potential brain abnormalities. QSM images were obtained from the complex mGRE image data via morphology‐enabled dipole inversion using an automatic uniform CSF zero‐reference algorithm (MEDI+0).^32^ The 3DT1W image processing was performed using VBM with diffeomorphic anatomical registration through exponentiated Lie algebra (DARTEL),^33^ based on the Statistical Parametric Mapping version 8.0 (SPM8) toolbox (http://www.fil.ion.ucl.ac.uk/spm/) of Matlab2014b software. The QSM images were segmented to the same resolution (1 × 1 × 2 mm^3^) as the gray matter volume images. To better match the anatomical characteristics of the sample population and achieve more accurate registration, the study‐specific brain template was generated as an average template from all subjects using the DARTEL algorithm.^33^ Then, QSM images were spatially normalized to the Montreal Neurological Institute (MNI) space, and smoothed with an isotropic Gaussian kernel of 3 mm full‐width at half‐maximum (FWHM). To avoid potential bias in susceptibility values across gray and white matter boundaries that may occur after smoothing, gray and white matter probability maps were derived from FreeSurfer segmentations generated via FreeSurfer (v6.0.1) within fMRIPrep pipeline and smoothed it separately to validate the previous results. Detailed processing steps are provided in the Supporting Information Figure S1.

### 2.5 Statistical analysis

All analyses were performed via SPSS version 26 and R software version 4.1.1 (University of Auckland, New Zealand) in both cohorts. A two‐sided *p* < 0.05 was considered statistically significant. Descriptive statistics such as the Kolmogorov‒Smirnov test and chi‐square test were used to compare baseline characteristics between Mets patients and HCs in both cohorts; the means and standard deviations were calculated for normally distributed variables. Two‐independent sample *t* tests were used to compare demographic, lifestyle, socioeconomic status, and susceptibility values between the two groups in both cohorts. Two‐independent sample *t* tests of SPM8 were used to compare the differences in susceptibility values between the two groups with age, gender, and years of education as covariates in Jinan cohort. Family‐wise error (FWE) correction was performed at the voxel‐level *p* < 0.001 and cluster‐level *p* < 0.05. The results were presented using xjview (http://www.alivelearn.net/xjview) and MRIcron software (https://github.com/neurolabusc/MRIcron), and RESTplus version 1.2 (http://www.restfmri.net) was used to extract the susceptibility values of the differential brain regions,^34^ referring to our previous study.^35^ Detailed processing steps are provided in the Supporting Information.

The exposure‒response relationships between Mets and the susceptibility values of the differential brain regions were investigated via three‐segment restricted cubic spline (RCS) in UKB. Pearson correlation analysis and multiple linear regression were used to analyze the correlation between Mets and the susceptibility values of the brain regions in both cohorts. The susceptibility values for each nucleus were used as dependent variables, and the relevant components of Mets were used as independent variables while controlling for the demographic, socioeconomic, and lifestyle covariates in UKB.

In longitudinal data, the adjusted volume was calculated as the ratio of the volume of the region of interest nucleus to the normalized gray matter volume. To avoid changes in gray matter volume over time, the estimated total iron content within the region of interest was defined as the product of the average susceptibility of each nucleus and its adjusted volume.^36^ The linear mixed model was used to analyze the age‐related changes of the Cau, putamen, pallidum, and thalamus in the two groups, with adjustments for covariates including demographic, socioeconomic, and lifestyle. Then, multiple linear regression was used with the cognitive test scores as dependent variable and the susceptibility values of differential brain regions as independent variables, after adjusting for age, sex, and education years in Jinan cohort.

We then performed subgroup analysis according to sex, age, smoking status, drinking status, ethnicity, and BMI in UKB and sex, age, smoking status, drinking status, and education in Jinan cohort. Details of the subgroup analysis can be found in the Supporting Information.

## 3 RESULTS

### 3.1 Basic characteristics of two cohorts

Table 1 presented the demographic, lifestyle, and socioeconomic characteristics of the Mets and HC groups in UKB and the Jinan cohort. The metabolic markers were obtained at the first visit to the UKB study, while the brain images used in this study were obtained after 8.8 ± 1.7 years. Participants in both cohorts were divided into two groups according to the Mets diagnostic criteria: the HCs group (8173 males, mean age of 53.78 ± 7.46 years in UKB; 60 males, mean age of 59.41 ± 8.46 years in Jinan cohort) and the Mets group (3663 males, mean age of 55.12 ± 7.35 years in UKB; 53 males, mean age of 59.93 ± 8.99 years in the Jinan cohort). The Mets group had more males, smokers, and drinkers in both cohorts and were older and lived in more socioeconomically deprived areas in UKB compared with the HCs group. The composition criteria of Mets in both cohorts were significantly different between the two groups. There were no significant differences in age, years of education, MoCA, TMT (A+B), and AVLT between the two groups in the Jinan cohort. The scores of SDMT and SCWT in the Mets group were significantly worse than those in the HCs group. Table S1 showed the demographic, lifestyle and socioeconomic characteristics of the Mets (294 participants, 175 males) and HCs (567 participants,191 males) groups with QSM image follow‐up data in UKB. In the longitudinal data, the mean age at the first MRI scan was 61.74 ± 7.39 for the Mets group and 58.77 ± 6.55 for the HCs group. At the follow‐up scan, the mean age was 64.42 ± 7.21 for the Mets group and 61.40 ± 6.37 for the HCs group. For detailed description, see the Supporting Information.

**TABLE 1:** Demographic and clinical characteristics of participants at the UKB and Jinan cohort.

Characteristic	Mets (*n* = 3663)	HCs (*n* = 17683)	*χ* ^2^/*t*	*p*‐Value
				Mets vs. HCs
**UKB**				
Age (*y*), mean (SD)	55.12 (7.35)	53.78 (7.46)	9.944	<0.001*^t^*
Sex, males *n* (%)	1953 (53.3%)	8173 (46.2%)	61.303	<0.001*^χ^* ^2^
Smoking, *n* (%)			25.008	<0.001*^χ^* ^2^
Never	2186 (59.7%)	11244 (63.6%)		
Previous	1237 (33.8%)	5388 (30.5%)		
Current	228 (6.2%)	1026 (5.8%)		
Missing	12 (0.3%)	25 (0.1%)		
Alcohol drinking, *n* (%)			19.398	<0.001*^χ^* ^2^
Never	104 (2.8%)	360 (2.0%)		
Previous	94 (2.6%)	316 (1.8%)		
Current	3464 (94.6%)	17003 (96.2%)		
Ethnic background			7.855	0.005
White	3415 (93.2%)	16243 (91.9%)		
Others	248 (6.8%)	1440 (8.1%)		
Townsend deprivation index, mean (SD)	−1.79 (2.75)	−1.94 (2.69)	2.980	0.003*^t^*
BMI index^2^, kg/m^2^, mean (SD)	30.16 (4.16)	25.45 (3.43)	63.775	< 0.001*^t^*
C‐reactive protein, mean (SD)	2.88 (3.57)	1.73 (3.17)	20.009	< 0.001*^t^*
HbA1c, mean (SD)	35.70 (4.43)	34.10 (3.48)	7.709	< 0.001*^t^*
Mets, *n* (%)				
Hyperglycemia	881 (24.1%)	1001 (5.7%)	1276.004	<0.001*^χ^* ^2^
Reduced HDL	2228 (60.8%)	2528 (14.3%)	3793.626	<0.001*^χ^* ^2^
Elevated triglycerides	3153 (86.1%)	4093 (23.1%)	5359.370	<0.001*^χ^* ^2^
Elevated blood pressure	3240 (88.5%)	8649 (48.9%)	1922.647	<0.001*^χ^* ^2^
Elevated waist circumference	2529 (69.0%)	2015 (11.4%)	6018.082	<0.001*^χ^* ^2^

Characteristic	Mets (*n* = 80)	HCs (*n* = 144)	*χ*2/*t*	*p*
				Mets vs. HCs
**Jinan cohort**				
Age (*y*), mean (SD)	59.93 (8.99)	59.41 (8.46)	−0.427	0.670*^t^*
Sex, males, *n* (%)	53 (66.3%)	60 (41.7%)	12.433	<0.001*^χ^* ^2^
Smoking, *n* (%)	29 (36.3%)	24 (16.7%)	10.919	0.001*^χ^* ^2^
Alcohol drinking, *n* (%)	43 (54.4%)	44 (30.8%)	11.955	0.001*^χ^* ^2^
Education (*y*), mean (SD)	13.03 (3.27)	13.35 (3.28)	0.720	0.472*^t^*
MoCA, mean (SD)	23.88 (3.77)	24.78 (2.25)	1.959	0.053 *^t^*
SDMT, mean (SD)	32.85 (13.73)	37.19 (13.45)	2.299	0.022 *^t^*
SCWT, mean (SD)	145.16 (43.78)	133.38 (28.04)	−2.172	0.032 *^t^*
TMT(A+B), mean (SD)	245.78 (126.57)	224.34 (107.95)	−1.323	0.187 *^t^*
AVLT, mean (SD)	55.97 (14.33)	56.86 (12.20)	0.479	0.632 *^t^*
HbA1c, mean (SD)	7.78 (1.82)	6.48 (1.70)	3.762	< 0.001*^t^*
Metabolic syndrome, *n* (%)				
Hyperglycemia	76 (95.0%)	51 (35.7%)	73.669	<0.001*^χ^* ^2^
Reduced HDL	15 (21.1%)	2 (1.4%)	25.442	<0.001*^χ^* ^2^
Elevated triglycerides	54 (74.0%)	23 (16.0%)	71.185	<0.001*^χ^* ^2^
Elevated blood pressure	61 (76.3%)	38 (27.0%)	50.164	<0.001*^χ^* ^2^
Obesity	66 (82.5%)	46 (31.9%)	52.578	<0.001*^χ^* ^2^

*Note*: *^χ^* ^2^Chi‐squared test, ^t^two independent sample *t*‐test;

Abbreviations: BMI, body mass index; HbA1c, hemoglobin A1c; HC, healthy controls; HDL, high‐density lipoprotein; Mets, metabolic syndrome; MoCA, Montreal cognitive assessment; SCWT, Stroop Color‐Word Test; SDMT, Symbol Digit Modalities Test; TMT: Trail Making Test; UKB, UK Biobank.

### 3.2 Associations between Mets and susceptibility values in deep gray matter nuclei

**FIGURE 2:**
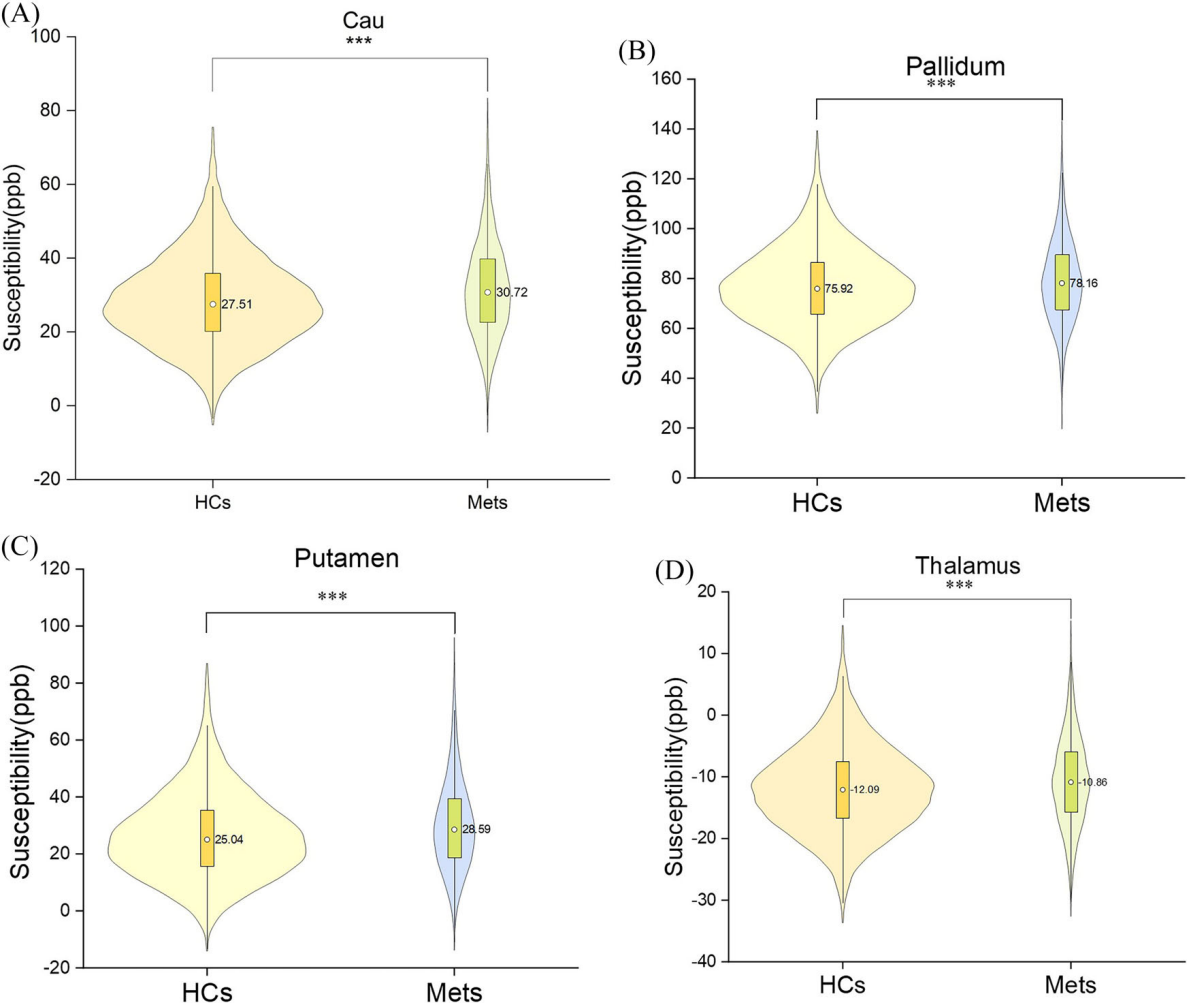
(A–D) Comparisons of susceptibility value in caudate nucleus (Cau), putamen, pallidum, and thalamus between Mets patients and HCs in UKB. The significance level of *p* values was Bonferroni corrected. ***p < 0.001. HC, healthy controls; Mets, metabolic syndrome.

The average susceptibility values of the Cau, pallidum, and putamen were positive in the two groups, and those of the thalamus were negative. The susceptibility values of the Cau, pallidum, and putamen and thalamus in the Mets group were significantly greater than those in the HCs group (all Bonferroni corrections *p* < 0.001) in UKB (Table 2 and Figure 2). In addition to the study objectives, we also found that the susceptibility values of the hippocampus and substantia nigra in the Mets group were significantly increased compared with HCs group, see Supporting Information Table S2. As shown in Figure S2, a linear relationship between Mets and the susceptibility values of the Cau, putamen, thalamus was observed in the RCS model. Therefore, as shown in Table S3 and Figure S3, we further explored the correlation of the susceptibility value of each nucleus with Mets score and composition criteria in UKB. The results revealed that the susceptibility values of the striatum, thalamus were positively correlated with Mets score (all *p* < 0.001). Multiple linear regression revealed that the susceptibility value in the Cau and putamen was mainly associated with elevated TGs (*β* = 2.01, *p* < 0.001; *β* = 2.39, *p* < 0.001) and hyperglycemia (*β* = 2.08, *p* = 0.006; *β* = 2.88, *p* = 0.001), whereas that in the pallidum was mainly associated with hyperglycemia (*β* = 2.13, *p* = 0.035) and in the thalamus (*β* = 0.65, *p* = 0.012) was mainly associated with elevated triglycerides (Table S3).

**TABLE 2:** Subgroup comparisons for susceptibility value in the in Mets and HCs in UKB.

Susceptibility value	Mets (*n* = 3663)	HCs (*n* = 17683)	*t*	*p*‐Value^#^
				Mets vs. HCs
Cau, mean (SD)	31.55 (12.99)	28.48 (12.18)	13.157	<0.001
Pallidum, mean (SD)	78.99 (16.90)	76.73 (16.13)	7.428	<0.001
Putamen, mean (SD)	30.18 (16.17)	26.46 (15.29)	12.794	<0.001
Thalamus, mean (SD)	−10.66 (7.29)	−11.90 (7.08)	9.374	<0.001

Abbreviations: Cau, caudate nucleus; HC, healthy controls; Mets, metabolic syndrome; SD, standard deviation; UKB, UK Biobank.

^#^ The Bonferroni method was used for correction.

Linear mixed model analysis using longitudinal data found that the susceptibility values of Cau and putamen in the two groups increased significantly with age (*β* = 0.58, *p* < 0.001; *β* = 0.54, *p* < 0.001), and the interaction effect between age and group of the two nuclei was significant (*β* = 0.16, *p* = 0.008; *β* = 0.25, *p* < 0.001), indicating that the changes of susceptibility values of Cau and putamen with age in the Mets group were significantly greater than those compared to the HCs group. Moreover, the interaction between age and group in the pallidum and thalamus was not significant, indicating no significant difference in the rate of increase with age between the two groups (Table S4).

**FIGURE 3:**
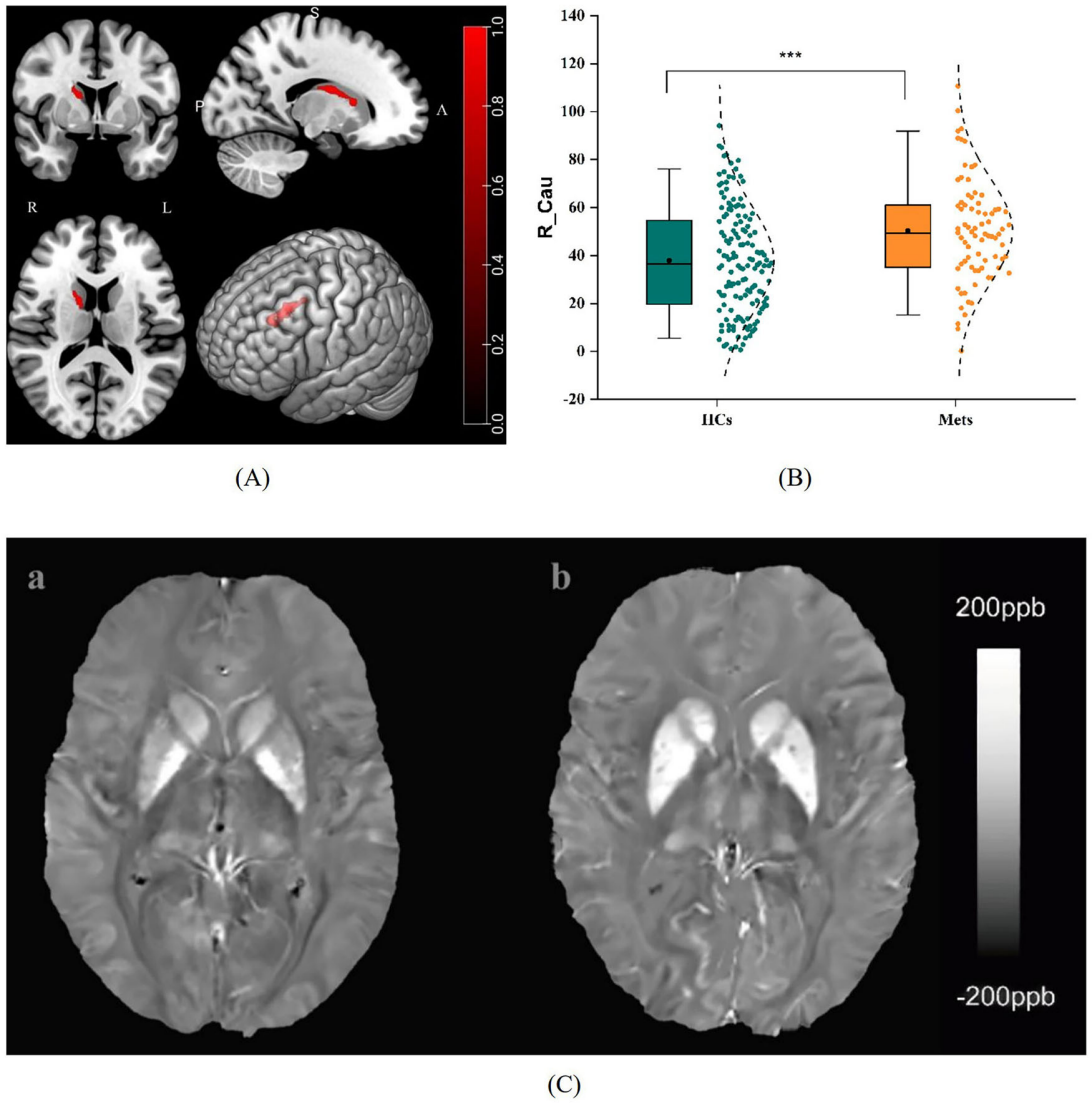
Whole‐brain analysis results of regional differences and box diagram between Mets patients and HCs in Jinan cohort. (A) Multiple section and three‐dimensional images made by MRIcron based on the result of VBM. Red: right caudate nucleus. (B) Susceptibility values of the right caudate nucleus between the two groups. (C) Differences in susceptibility values between the two groups. The images are example participants from the two groups (a: HC, a 66‐year‐old male; b: a Mets patient, a 67‐year‐old male). ***:*p* < 0.001. HCs, healthy controls. Mets, metabolic syndrome.

As shown in Tables 3, S5 and Figure 3, the susceptibility value of the right caudate nucleus (R_Cau) was significantly increased in the Mets group compared with the HCs group after FWE correction (peak MNI: 13, 1, 16; *T* = 4.76, FWE‐corrected *p* = 0.006) (peak MNI: 16, 3, 15; *T* = 4.61, FWE‐corrected *p* = 0.005) in Jinan cohort. We further revealed that the susceptibility value of R_Cau was positively correlated with Mets score after adjusting for age, sex, and years of education (*β* = 1.56, *p* = 0.003) (Table S6).

**TABLE 3:** Brain regions with significantly altered susceptibility values between the two groups of whole brain QSM images in Jinan cohort.

Condition	Clusters	Cluster voxels	Peak MNI			*T*	*Z*	*p*‐Value^#^
			X	Y	Z			
Mets > HCs	R_Cau	774	13	1	16	4.76	4.64	0.006

*Note*: Cluster size: the number of voxels in the identified significant cluster.; MNI: Montreal neurological institute;.

Abbreviations: Cau, caudate nucleus; HC: healthy controls; Mets: metabolic syndrome; MNI, Montreal Neurological Institute; QSM, quantitative susceptibility mapping; R_Cau: right caudate nucleus.

^#^ The family‐wise error (FWE) method was used for correction.

### 3.3 Associations between susceptibility values of R_Cau and cognitive function in Jinan cohort

**FIGURE 4:**
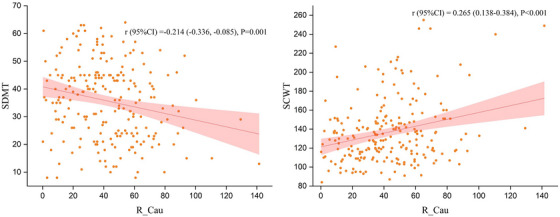
Associations between susceptibility values of right caudate nucleus and cognitive function of all participants in Jinan cohort. SDMT, symbol digit modalities test; SCWT, Stroop color‐word test; R_Cau, right caudate nucleus. Susceptibility value [ppb(*10^−9^)]. *r*: Pearson correlation coefficient. *p*: *p*‐value of Pearson correlation analysis.

The Pearson correlation analysis results of the susceptibility values of R_Cau with SDMT and SCWT in the cohort are shown in Figure 4. Multiple linear regression results, adjusted for age, sex, and education, demonstrated that the susceptibility value of R_CAU was significantly positively correlated with SCWT (*β* = 0.242, *p* = 0.003) in the cohort (Table 4). After adjustment for covariates, the susceptibility value of R_Cau was not significantly associated with SDMT. The relationship between the R_Cau and SCWT and SDMT in the HCs and Mets groups was shown in the Supporting Information (Table S7).

**TABLE 4:** Multivariate analysis results of the associations between susceptibility values in right caudate and cognitive function.

Cognitive test	Variables	*β* (95% CI)	*t*	*p*‐Value	*R*^2	*p*‐Value
SCWT	R_Cau	0.242 (0.082, 0.402)	2.983	0.003	0.273	<0.001
	Age	0.664 (0.160, 1.168)	2.595	0.010		
	Gender	−16.929 (‐27.784, ‐6.073)	−3.074	0.002		
	Education	−3.635 (‐4.977, ‐2.293)	−5.339	<0.001		
SDMT	Age	−0.803 (‐0.951, ‐0.654)	−10.661	<0.001	0.599	<0.001
	Education	1.551 (1.157,1.946)	7.743	<0.001		

Abbreviations: CI, confidence interval; MoCA, Montreal Cognitive Assessment; R_Cau, right caudate nucleus; SCWT: Stroop Color‐Word Test; SDMT, Symbol Digit Modalities Test.

### 3.4 Subgroup analysis

As shown in Figure S4, the mean susceptibility values of striatum and thalamus were significantly associated with Mets score in all age, sex, ethnic, and BMI groups in UKB. The susceptibility values of R_Cau were significantly associated with Mets score in all age, sex, and educational level in the Jinan cohort. Significant correlations were found regardless of whether the subjects smoked or drank alcohol in both cohorts. Nevertheless, the susceptibility value of R_Cau was significantly positively correlated with SCWT in elderly males, smokers and drinkers, and participants with less than 16 years of education in Jinan cohort.

## 4 DISCUSSION

This study capitalized two independent cohorts from UK and Chinese populations to respectively explore the relationships among Mets, brain iron, and cognitive function. The results demonstrated that the regional brain iron deposition might serve as a neuroimaging marker of Mets. A significant association was found between brain iron deposition and cognitive performance. Our results provided new insights into Mets‐related brain damage and its relationship with cognitive decline.

First, based on the large sample database of middle‐aged and elderly people without major diseases, we determined that increased susceptibility values in the striatum and thalamus are associated with Mets, which is consistent with existing studies on Mets‐related brain damage. The thalamus exhibits a unique cellular architecture interspersed with white matter fiber bundles and myelinated systems,^37^ resulting in an anti‐magnetic signal with a negative susceptibility value relative to CSF. Nevertheless, the thalamus is a gray matter dominated structure that has been reported to exhibit iron accumulation in various neurodegenerative diseases.^38^, ^39^, ^40^ The susceptibility values of striatum and thalamus in Mets patients were significantly increased in cross‐sectional studies, and the susceptibility values of striatum in the Mets group increased greater with age than those compare to HCs in longitudinal studies, suggesting that Mets may accelerate the accumulation of brain iron. A systematic review addressed the link between brain iron and Mets and type 2 diabetes mellitus (T2DM) while discussing similarities in Alzheimer disease (AD) mechanisms in human subjects and animal models.^41^ Previous studies have shown a significant correlation between Mets severity and right caudate nucleus volume.^20^ Afterwards, we conducted a voxel‐based analysis of QSM in the Jinan cohort and found that the brain iron in the right caudate nucleus (R_Cau) was significantly increased. Brain iron deposits in deep gray matter nuclei of Mets patients, especially in the caudate nucleus, were found in both cohorts. Currently, the relationship between Mets and brain iron imbalance and its mechanisms are being explored.

The main pathophysiological changes in Mets may include endothelial dysfunction, chronic oxidative stress, and systemic inflammation.^42^ Oxidative stress caused by metabolic overload can activate the inflammatory response, leading to systemic chronic low‐grade inflammation and elevated levels of proinflammatory factors such as tumor necrosis factor‐α (TNF‐α), interleukin‐6 (IL‐6), and IL‐18, thereby promoting damage to vascular endothelial cells and neuronal cells.^43^, ^44^ In addition, the increase in the brain iron concentration may be caused by increased inflammation, increased blood–brain barrier permeability and redistribution of brain iron.^45^ Neuroinflammation in the central nervous system is mediated mainly by specific cell types, such as microglia, astrocytes, and endothelial cells. However, in the case of impaired brain–blood barrier integrity, peripheral inflammatory cells, such as macrophages, may also exacerbate inflammation. Glial cells are activated, and the iron balance is disturbed as neuroinflammation occurs. In vitro studies have shown that short‐term stimulation may increase iron accumulation in neurons and microglia via TNF‐α and IL‐6.^46^ Iron accumulation in neurons may induce brain damage through apoptosis. Microglia can promote the regeneration and repositioning of damaged neurons through synaptic pruning. However, brain microglia remain chronically activated during neuroinflammation and can produce reactive oxygen species (ROS), inducing oxidative stress through multiple signaling pathways.^47^, ^48^ Meanwhile, Mets‐induced brain iron deposition showed regional heterogeneity, which might be related to the altered microglial and astrocytes function in the striatum and thalamus, that is, Mets accelerate brain iron deposition in these regions. In Jinan cohort, iron deposition in Mets population with relatively small sample was more concentrated in R_Cau, which might be due to the differences in race, sample size, the inherent lateralization function of the brain and the selectivity of metabolic stress. Previous studies have shown that Mets patients are often accompanied by behavioral problems such as seeking high‐sugar and high‐fat foods, which may be closely related to dopamine striatal system dysfunction.^49^, ^50^ The R_Cau plays a key role in the impulse–reward circuit as an important component of the striatum, and dopaminergic activity related to reward and motivation may exhibit a rightward dominance.^51^, ^52^ Additionally, all subjects in our study were right‐handed, with the right hemisphere generally dominating in impulse control and related functions.^53^ This may have placed the right hemisphere under higher functional burden, rendering it more vulnerable to inflammatory‐mediated damage.^54^

However, it is still controversial whether the different components of Mets each form a distinct pathology or belong to a common, broader pathogenic process. We further explored the metabolic factors closely related to the nuclei and found that the increased susceptibility values of the Cau and putamen were closely related to the elevated blood glucose and triglyceride. In addition, the increased susceptibility values of the pallidum and thalamus were associated with elevated blood glucose and triglyceride, respectively. While several studies have shown that the functional connectivity of the frontostriatal circuit in patients with T2DM was abnormal,^55^ the brain iron in deep gray matter nuclei involved in movement and cognition in T2DM was increased.^56^ Previous studies have shown that triglycerides and glucose can be used as markers of IR in healthy adults.^57^ A study of brain iron overload and IR in obese subjects revealed that obesity and IR may increase liver and brain iron concentrations and are associated with cognitive decline.^8^ Specifically, iron overload in the caudate nucleus had the highest sensitivity and specificity in predicting obesity. It is reasonable to speculate that in IR, the targeted brain regions suffer iron excess due to high reactivity and the generation of hydroxyl radicals, which trigger increased oxidative stress and subsequent functional impairment.^58^ IR also appears to promote ROS formation by promoting the formation of advanced glycation end products, which may lead to increased iron deposition in the striatum and affect its normal function.^59^ Previous studies in hypertensive patients have shown that brain iron deposition occurs mainly in the Cau, putamen, and dorsal thalamus.^60^ Our findings revealed new insights that suggest some potential practical interventions for clinical practice. Aggressive interventions that target high‐risk factors closely related to brain iron deposition could be helpful. Approaches like geographic mapping of people at risk of hyperglycemia, hyperlipidemia and increasing screening and treatment services for socially vulnerable groups would be significant.

Furthermore, our data indicated that iron deposition in the R_Cau was associated with cognition, especially executive function and attention control. Accumulating evidence suggested that Mets patients performed poorly in cognitive tests of working memory, processing speed, attention, and executive function.^61^, ^62^ Previous studies have shown that iron deposition in the basal ganglia is related to cognitive function impairment,^11^, ^63^ especially slower executive function and lower fluid intelligence.^64^ Normal iron homeostasis is crucial for maintaining optimal brain function. The selective accumulation of iron in specific brain regions causes intracellular redox imbalance, lipid peroxidation, and mitochondrial dysfunction,^65^ which affects cognitive function and even increasing the susceptibility to future neurodegenerative diseases.^66^ Subgroup analysis revealed that the mean susceptibility value of the striatum and thalamus among people of different ages, genders, races, and BMI in the UKB was significantly correlated with the Mets score. This significant correlation also existed in different populations of Jinan cohort. Notably, the correlation between susceptibility value of R_Cau and SCWT score was more significant in elderly men, smokers and drinkers, and people with less than 16 years of education in the Jinan cohort. The available evidence suggested that we should pay special attention to the control of metabolic risk factors such as blood glucose and obesity in elderly men and people who smoke and drink.

The strengths of this study are to apply two ethnic databases to corroborate each other. First, the UKB with sufficient sample size was used for initial exploration, and then further exploration was carried out in the relatively small sample cohort. Our study has several limitations. First, the cross‐sectional design limits the exploration of causality, and future studies will continue to explore image data from longitudinal follow‐up of participants. Second, we did not investigate changes in the susceptibility value of the corticostriatal circuit in Mets participants because of the absence of white matter susceptibility values in the UKB. Similarly, no significant difference in cortical susceptibility values was found in Mets patients in Jinan cohort. The effect on the cortical susceptibility value will continue to be investigated in our study cohort in the future.

## 5 CONCLUSIONS

In this study, we provide evidence that Mets can contribute to iron deposition in the putamen, Cau, pallidum, and thalamus in UKB and R_Cau in Jinan cohort. Our findings suggested that iron deposits in the R_Cau were associated with cognitive dysfunction in Jinan cohort. Regional brain iron deposition may be a neuroimaging biomarker of the severity of Mets, providing new insights into Mets‐related brain injury and its relationship with cognitive decline.

## FUNDING

This work was supported by the Natural Science Foundation of Shandong Province (ZR2020MH288, ZR2024MH026), the Technology Development Plan of Jinan (202328066), Funding for Study Abroad Program by Shandong Province (201803059), and the Medical and Health Science and Technology Development Project of Shandong Province (202309010557; 202309010560; 202409010479), and the Shandong Province Medical System Employee Science and Technology Innovation Plan (SDYWZGKCIH2023034; SDYWZGKCIH2024021).

## CONFLICT OF INTEREST STATEMENT

The authors declare that this study was conducted in the absence of any commercial or financial relationships that could be construed as a potential conflict of interest. Author disclosures are available in the Supporting Information.

## CONSENT STATEMENT

The UK Biobank protocol was approved by the NHS North West Multicentre Research Ethics Committee (11/NW/0382). All human subjects provided informed consent in both cohorts.

## Supporting information



Supporting Information

Supporting Information
