# Supplementary material for: Associations between metabolic syndrome and regional brain iron depositions and cognitive function in middle‐aged and older adults: A two multinational cohort study
Source: Alzheimers Dement. 2026 Jan 8;22(1):e71080. doi: 10.1002/alz.71080 (PMC12782154; doi:10.1002/alz.71080)
Supplement: Supplementary file 1 — Supporting Information [file ALZ-22-e71080-s001.pdf]

2. Materials and methods

2.1 Participants

We included participants with susceptibility-weighted MRI (SWI) data (n = 41638) and removed participants due to (1) missing Mets data (n = 3339); (2) a history or current diagnosis of any cancer, cerebral trauma, cardiovascular, neurologic, or psychiatric disorders (n=15810); or (3) a history or current substance abuse or dependency (n=33) for a total of 22456 participants after exclusion. We then used the Mahalanobis distance method to exclude 1110 subjects with outliers in the susceptibility value. In the Jinan cohort, approximately 522 individuals collected MRI brain imaging data, as well as other health-related data. We included adults aged 40 to 70 years old (n=385) and excluded participants with incomplete data (n=144) and self-reported or hospital-recorded history of dementia or other serious neurological disorders (n=5).

2.2 Metabolic syndrome

A Beckman Coulter AU5800 analyzer was used to measure triglyceride and HDL levels in blood serum. Omron 7015IT monitors were used to measure blood pressure. Waist circumference was measured via a Seca 200 measuring tape, with participants removing bulky clothing beforehand.

2.3 Cognitive assessment

The TMT consists of two parts (TMT-A and TMT-B). The subjects were asked to connect randomly arranged circle numbers containing 1 to 25 in sequence as quickly as possible in TMT-A. TMT-B is similar with TMT-A, but subjects must alternate between

numbers and letters. We use the sum of the time taken to complete the two tasks (TMT-B+TMT-A) as the judgment score. In SCWT, the subjects were asked to read three different forms as quickly as possible. Two of the tables represented "consistent conditions," in which subjects were asked to read the colors printed in black ink and the names of different color blocks. Instead, in the third table, the color word is printed with inks of inconsistent colors. The sum of time spent reading the three forms was used as the judgment score. In SDMT, the nine symbols correspond to the numbers 1-9. The lines to be answered contain only symbols, and the subject's task is to write or verbally report the correct number corresponding to the symbols in the following spaces. After completing the first 10 items under instruction, record how many responses the subject can complete in 90 seconds. The AVLT contains 12 words that are read three times in a row. Each reading was followed by a free recall test. Then, with a delay of 5 and 20 minutes, the subjects were asked to freely recall the 12 words again. Finally, recognition memory was tested using a 24-word disturbance list. The sum of the last six memorized words is used as the judging score.

2.4 MRI acquisition and processing

The final quantitative susceptibility mapping (QSM) pipeline for UK Biobank susceptibility-weighted imaging (SWI) data is as follows. (1) To generate the phase map of the images, it is first necessary to combine the phase images from the individual coil channels. The pipeline employs the MCPC-3D-S method to combine phase images across channels [1], generating channel-combined phase images for each echo without

any phase cancellation artifacts. (2) The phase image of the channel combination is unwrapped using the Laplace-based algorithm provided by the STI Suite toolkit [2]. (3) Subsequently, the unwrapped phase images are merged into a single phase dataset by weighting the two echoes [3], thereby enhancing the signal-to-noise ratio. Subsequently, the V-SHARP algorithm within the STI Suite toolbox was employed to filter the echo-combined phase data [4], thereby removing the background field contributions, with a maximum kernel size of 12 mm. (4) QSM was generated from V-SHARP filtered phase data using the refined brain mask. Dipole inversion was performed using the iLSQR algorithm from the STI Suite toolbox [5].

To estimate the overall magnetic field, the multi-echo data was first fitted nonlinearly. The local field was then calculated by unwrapping the spatial field and removing the background field using the projection onto dipole fields (PDF) algorithm. The final susceptibility map was then created by inverting the local field. Structural priors (edges) from the magnitude picture were included in the numerical inversion along with a regularization term to ensure uniform CSF susceptibility in the lateral ventricles. This improved the QSM's accuracy and provided an automatic susceptibility reference. With voxel connectivity limitations, the R2* map obtained from the mGRE magnitude data was thresholded to create the CSF mask[6]. We used a strict quality control procedure in the Jinan cohort. All segmented images were visually inspected and validated by LF Guo, a neuroradiologist with over 20 years of experience, to ensure data accuracy and consistency.

2.5 Statistical analysis

In the Jinan cohort, we initially utilized the SPM8 toolbox in Matlab2014b software to perform a two-independent samples t-test to identify significantly different clusters between the two groups. Subsequently, xjview (http://www.alivelearn.net/xjview) was used for result visualization and extraction of regions of interest (ROIs), with the coordinates of the regions of ROI. We then utilized the Extract ROI Signals within the RESTplus version 1.22 (http://www.restfmri.net) utilities to extract the mean susceptibility values for the two groups of ROIs for subsequent correlation analysis. MRIcron software (https://github.com/neurolabusc/MRIcron) was used for image visualization of results.

We conducted a subgroup analysis of the correlation between the average susceptibility values of different brain regions and Mets score in both cohorts and the correlation between the susceptibility values of different brain regions and cognitive function. Smoking status and drinking status were classified to never, previous or current in UKB. BMI was graded according to World Health Organization standards.

3. Results

3.1 Basic characteristics of two cohorts

Among the participants with follow-up data, the Mets group was older, had a higher proportion of males, and exhibited higher body mass index (BMI) and C-reactive protein levels compared to the HCs group. There were no significant differences in

smoking and alcohol drinking status, ethnicity and socioeconomic indicators between the two groups.

3.3 Associations between susceptibility values of R_Cau and cognitive function in Jinan cohort

The susceptibility value of R_Cau was significantly positively correlated with SCWT (β=0.202, P=0.037) in the HCs group but no significant correlation was found in the Mets group (Table S6).

Table S1. Demographic and clinical characteristics of participants with longitudinal data from the UK Biobank.

**Characteristic**	**Mets (n=294)**	**HCs (n=567)**	**χ^2 /t**	**P**
				Mets vs. HCs
**Age 1 (y), Mean (SD)**	61.74 (7.39)	58.77 (6.55)	5.802	<0.001^t
**Age 2 (y), Mean (SD)**	64.42 (7.21)	61.40 (6.37)	6.051	
**Sex, males n (%)**	175 (59.5%)	191 (33.7%)	52.888	<0.001$^{\chi 2}$
**Smoking, n (%)**			6.786	0.079$^{\chi 2}$
Never	178 (60.5%)	387 (68.3%)		
Previous	98 (33.3%)	152 (26.8%)		
Current	17 (5.8%)	28 (4.9%)		
Missing	1 (0.3%)	0 (0%)		
**Alcohol drinking, n (%)**			0.072	0.965$^{\chi 2}$
Never	4 (1.4%)	9 (1.6%)		
Previous	5 (1.7 %)	10 (1.8%)		
Current	285 (96.9%)	548 (96.6%)		
**Ethnic background**			1.456	0.228
White	273 (92.9%)	538 (94.9%)		
Others	21 (7.1%)	29 (5.1%)		
**Townsend deprivation index, Mean (SD)**	- 1.78 (2.99)	-1.89 (2.67)	0.532	0.595^t
**BMI index^2, kg/m^2, Mean (SD)**	29.78 (5.41)	23.53 (3.60)	17.867	< 0.001^t
**C-reactive protein, Mean (SD)**	2.65 (2.87)	1.18 (2.50)	7.441	< 0.001^t

Note: Age1: Age at first scan; Age 2: Age at follow-up scan; $^{\chi 2}$Chi-square test, ^{t}two independent sample t-test; HbA1c, hemoglobin A1c; HDL, high-density lipoprotein; Mets: metabolic syndrome; HCs: healthy controls.

Table S2. Subgroup comparisons for susceptibility value in Mets and HCs in UKB.

**Susceptibility value**	**Mets (n=3663)**	**HCs (n=17683)**	**t**	**P#**
				**Mets vs. HCs**
**Accumbens, Mean (SD)**	-7.46 (12.99)	-7.35 (8.73)	-0.598	0.550
**Amygdala, Mean (SD)**	-8.29 (7.85)	-8.12 (7.09)	-1.081	0.373
**Hippocampus, Mean (SD)**	-5.14 (6.08)	-6.77 (6.06)	13.569	<0.001
**Substantia nigra, Mean (SD)**	65.40 (15.89)	64.24 (15.57)	3.749	<0.001

Note: Mets: metabolic syndrome; HCs: healthy controls; Cau: caudate nucleus. #The false discovery rate correction method was used for correction.

Table S3. Multivariable linear regression analyses examining association between the components of Mets and susceptibility value in the striatum and thalamus in UKB.

		Mets score	Hyperglycemia	Reduced HDL	Elevated triglycerides	Elevated blood pressure	Elevated waist circumference
**Cau**	**β**	1.52	2.08	0.39	2.01	0.66	0.85
	**t**	9.13	2.76	0.77	4.41	1.49	1.30
	**P**	<0.001	0.006	0.442	<0.001	0.136	0.193
	**95%CI**	1.19 to 1.84	0.60 to 3.55	-0.60 to 1.37	-11.74 to 1.68	-0.21 to 1.53	-0.43 to 2.14
**Pallidum**	**β**	1.28	2.13	0.66	1.02	-0.18	-1.04
	**t**	5.82	2.11	0.98	1.67	-0.30	-1.19
	**P**	<0.001	0.035	0.329	0.094	0.766	0.234
	**95%CI**	0.85 to 1.71	0.15 to 4.11	-0.66 to 1.98	-0.17 to 2.22	-1.34 to 0.99	-2.77 to 0.68
**Putamen**	**β**	1.38	2.88	0.23	2.39	1.54	1.41
	**t**	6.98	3.19	0.38	4.38	2.91	1.45
	**P**	<0.001	0.001	0.703	<0.001	0.004	0.147
	**95%CI**	0.99 to 1.77	1.11 to 4.65	-0.95 to 1.41	1.32 to 3.47	0.50 to 2.58	-0.40 to 2.68
**thalamus**	**β**	0.72	-0.01	0.31	0.65	0.40	0.24
	**t**	7.56	-0.02	1.08	2.51	1.57	0.63
	**P**	<0.001	0.988	0.282	0.012	0.116	0.529
	**95%CI**	0.54 to 0.91	-0.85 to 0.83	-0.25 to 0.87	0.14 to 1.16	-0.10 to 0.89	-0.50 to 0.97

Note: Cau: caudate nucleus.; Mets: metabolic syndrome; HDL, high-density lipoprotein; The model adjusted for age, sex, ethnicity, Townsend deprivation index, alcohol drinking, smoking status.

Table S4. Linear mixed-effects model results for the susceptibility value in the striatum and thalamus in HC and Mets groups from longitudinal data in UKB.

	Variables	Estimate	SE	95%CI-low	95%CI-high	P
**Cau**	**Age**	0.58	0.05	0.48	0.68	<0.001
	**Group**	1.42	0.43	0.58	2.26	0.001
	**Age*Group**	0.16	0.06	0.04	0.28	0.008
	**Mets score**	0.09	0.02	0.05	0.13	<0.001
**Pallidum**	**Age**	1.15	0.07	1.01	1.29	<0.001
	**Group**	1.93	0.59	0.77	3.09	0.001
	**Age*Group**	0.06	0.08	-0.10	0.22	0.453
	**Mets score**	0.15	0.03	0.09	0.21	<0.001
**Putamen**	**Age**	0.54	0.05	0.44	0.64	<0.001
	**Group**	1.75	0.40	0.97	2.53	<0.001
	**Age*Group**	0.25	0.05	0.15	0.35	<0.001
	**Mets score**	0.08	0.02	0.04	0.12	<0.001
**thalamus**	**Age**	-0.30	0.02	-0.34	-0.26	<0.001
	**Group**	-0.48	0.20	-0.87	-0.09	0.016
	**Age*Group**	-0.02	0.03	-0.08	0.04	0.502
	**Mets score**	-0.04	0.77	-0.06	-0.02	<0.001

Note: Cau: caudate nucleus.; Mets: metabolic syndrome; The model adjusted for age, sex, ethnicity, Townsend deprivation index, alcohol drinking, smoking status.

Table S5. Brain regions with significantly altered susceptibility values between the two groups of gray matter QSM images in Jinan cohort.

Condition	Clusters	Cluster voxels	Peak MNI			T	Z	P#
			X	Y	Z			
**Mets>HCs**	R_Cau	665	16	3	15	4.61	4.50	0.005

Note: HCs: healthy controls; Mets: metabolic syndrome; R_Cau: right caudate nucleus; Cluster size: the number of voxels in the identified significant cluster.; MNI: Montreal Neurological Institute; #The familywise error (FWE) method was used for correction.

Table S6. Univariate and multivariate analysis results of the associations between Mets score and susceptibility values in right caudate nucleus.

Cluster	Variables	Univariate				Multivariate			
		β	t	P	95%CI	β	t	P	95%CI
**R_Cau**	**Age**								
	**Male**								
	**0**				0.00 (Reference)				0.00 (Reference)
		-5.03	-0.10	0.141	-11.74 to 1.68	-7.23	-0.14	0.888	-10.83 to 9.38
	**Smoking**								
	**0**				0.00 (Reference)				0.00 (Reference)
	**1**	8.12	2.03	0.043	0.25 to 15.98	4.41	0.84	0.403	-5.97 to 14.78
	**Alcohol drinking**								
	**0**				0.00 (Reference)				0.00 (Reference)
	**1**	2.59	0.74	0.458	-4.28 to 9.47	-3.88	-0.80	0.428	-13.50 to 5.74
	**Education**	-0.66	-1.27	0.207	-1.69 to 0.37	0.04	0.07	0.945	-1.16 to 1.24
	**Mets score**	1.56	2.82	0.005	0.47 to 2.65	4.58	3.00	0.003	1.57 to 7.59

Note: R_Cau: right caudate nucleus.

Table S7. Multivariate analysis results of the associations between susceptibility values in right caudate and cognitive function in the HCs and Mets groups.

Group	Cognitive test	Variables	β (95% CI)	t	P	R^2	P
**HCs**	**SCWT**	**R_Cau**	0.202 (0.012, 0.391)	2.103	0.037	0.360	<0.001
		**Age**	0.457 (-0.091, 1.005)	1.649	0.010		
		**Gender**	-21.997 (-30.389, -13.604)	-5.186	<0.001		
		**Education**	-3.263 (-4.608, -1.919)	-4.803	<0.001		
	**SDMT**	**R_Cau**	-0.024 (-0.096, 0.048)	-0.661	0.510	0.552	<0.001
		**Age**	-0.668 (-0.873, -0.463)	-6.446	<0.001		
		**Gender**	5.299 (2.127, 8.470)	3.304	0.001		
		**Education**	1.740 (1.226, 2.254)	6.699	<0.001		
**Mets**	**SCWT**	**R_Cau**	0.253 (-0.102, 0.608)	1.421	0.159	0.247	<0.001
		**Age**	1.004 (-0.060, 2.068)	1.880	0.064		
		**Gender**	-21.091 (-40.717, -1.466)	-2.141	0.036		
		**Education**	-4.694 (-7.729, -1.659)	-3.081	0.003		
	**SDMT**	**R_Cau**	-0.027 (-0.102. -0.049)	-0.707	0.482	0.655	<0.001
		**Age**	-1.005 (-1.231, -0.779)	-8.866	<0.001		
		**Gender**	3.725 (-0.440, 7.889)	1.782	0.079		
		**Education**	1.254 (0.610, 1.898)	3.880	<0.001		

Note: HCs: healthy controls; Mets: metabolic syndrome; R_Cau: right caudate nucleus; SCWT: stroop color -word test; SDMT: symbol digit modalities test.

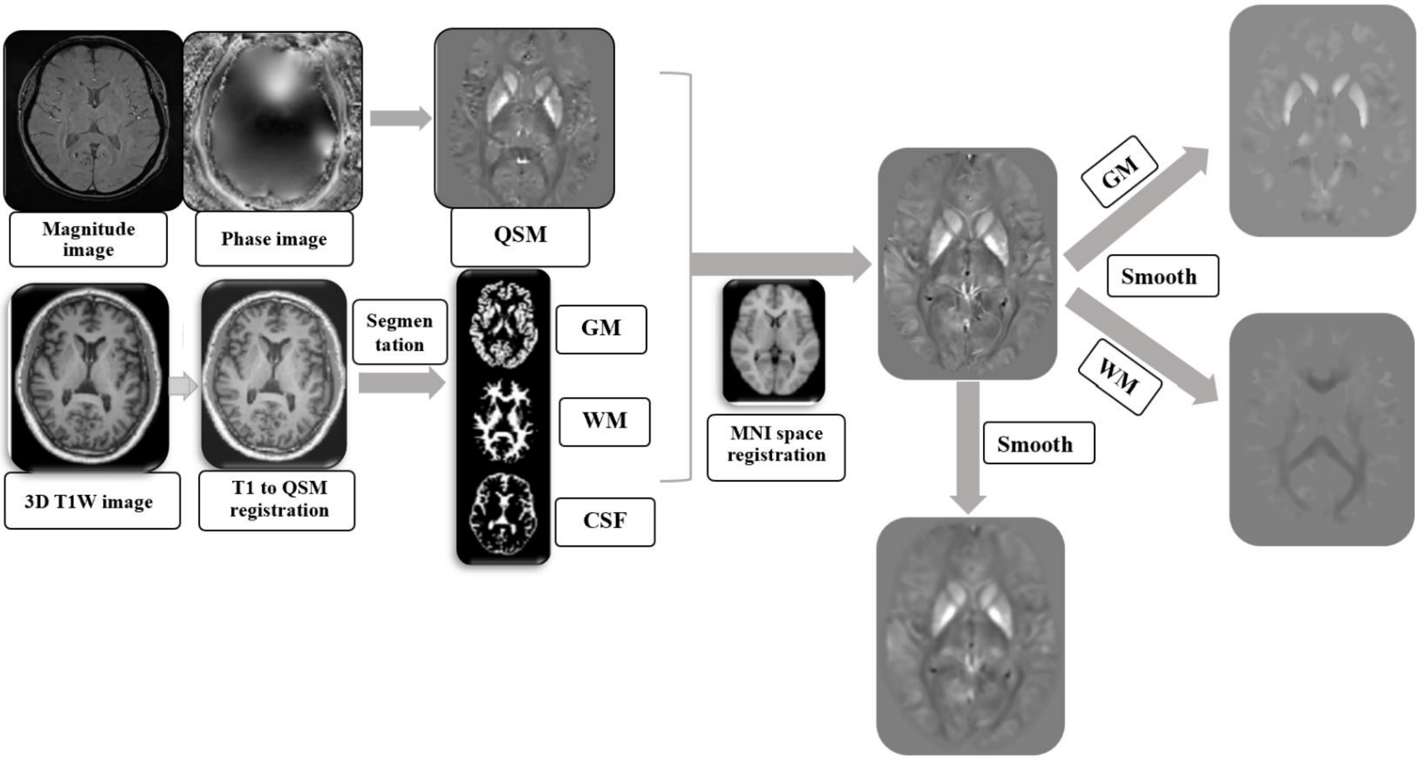

Figure S1. Flowchart of QSM image preprocessing. QSM: Quantitative susceptibility mapping; GM: Gray matter; WM: White matter; CSF: Cerebrospinal fluid; MNI: Montreal neurological institute. 3D T1W: Three-dimensional T1-weighted.

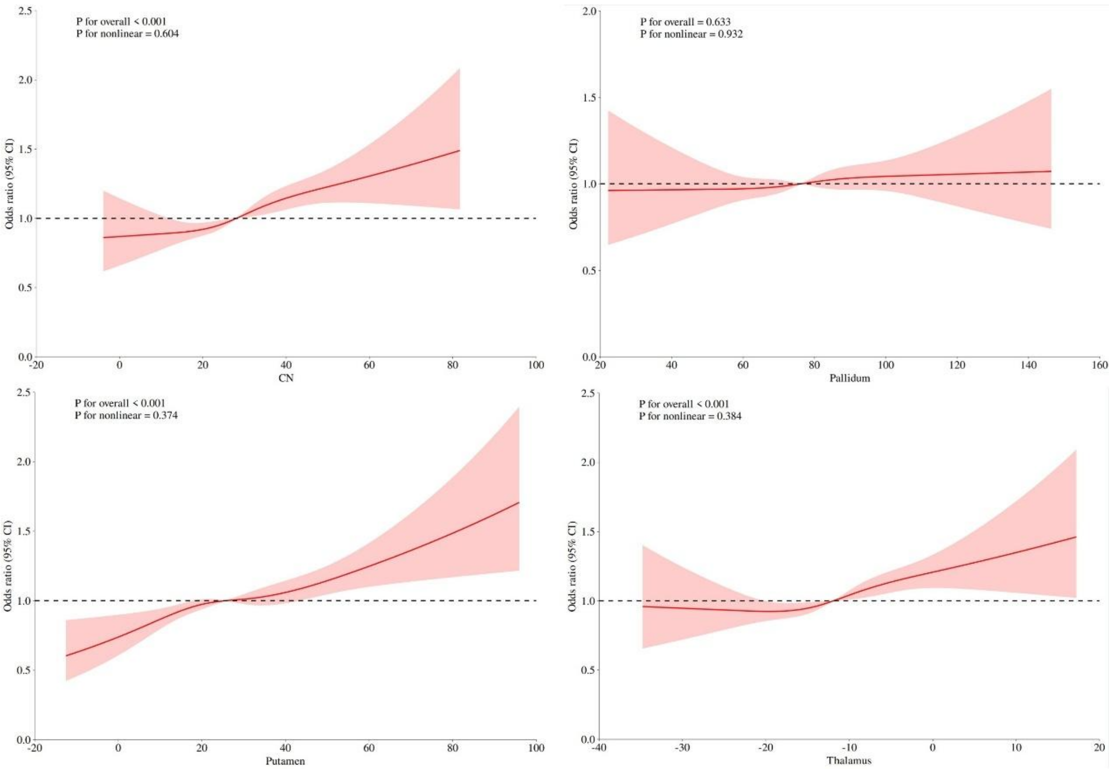

Figure S2. The exposure-response curve relations between caudate nucleus (CN), putamen, pallidum, and thalamus susceptibility values and metabolic syndrome (model adjusted for sex, age, ethnicity,

Townsend deprivation index, alcohol drinking, smoking status, body mass index and C-reactive protein). A restricted cubic spline was fitted to model each curve, with 4 knots fixed at the 5th, 35th, 65th, and 95th percentiles for all smooth curves. P values for nonlinearity were calculated using the Wald chi-square test.

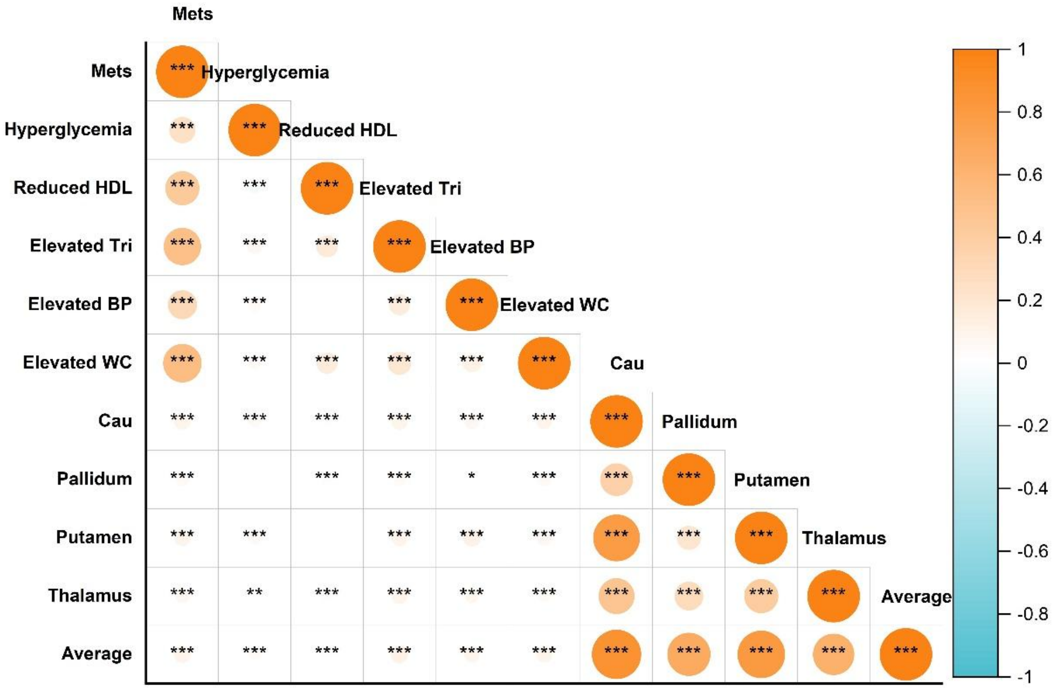

Figure S3. Heat maps of Mets and their components in relation to susceptibility value in caudate nucleus, putamen, pallidum and thalamus in UKB. The numerical values on the bar graph on the right side of the figure represent the Pearson correlation coefficient (r value). The size and color intensity of the circles represent the magnitude and direction of the corresponding r value. *: P<0.05; **: P<0.01; ***:P<0.001. Mets: metabolic syndrome; Cau: caudate nucleus; HDL: high-density lipoprotein; Tri: triglycerides; BP: blood pressure; WC: waist circumference.

		n (%)	OR	95%LCI	95%UCI	P
Age	40-59	15498 (72.60)	0.67	0.51	0.82	<0.001
	60-70	5848 (27.40)	0.55	0.28	0.82	<0.001
Sex	Male	10126 (47.44)	0.58	0.40	0.77	<0.001
	Female	11220 (52.56)	0.69	0.50	0.89	<0.001
Smoking	Never	13430 (63.03)	0.58	0.41	0.74	<0.001
	Previous	1254 (5.88)	0.79	0.23	1.34	0.006
	Current	6625 (31.09)	0.65	0.41	0.89	<0.001
Drinking	Never	464 (2.17)	1.36	0.45	2.26	0.004
	Previous	410 (1.92)	0.14	-0.78	1.06	0.768
	Current	20467 (95.91)	0.62	0.48	0.75	<0.001
Ethnic	White	19658 (92.09)	0.62	0.48	0.75	<0.001
	Others	1688 (7.91)	0.72	0.24	1.20	0.003
BMI	<18.5	93 (0.44)	1.27	0.34	2.20	0.008
	18.5-24.9	8572 (40.60)	0.51	0.27	0.75	<0.001
	25-29.9	9080 (43.00)	0.62	0.43	0.81	<0.001
	30-34.9	2721 (12.89)	0.51	0.15	0.86	0.005
	>35	648 (3.07)	0.93	0.16	1.69	0.017

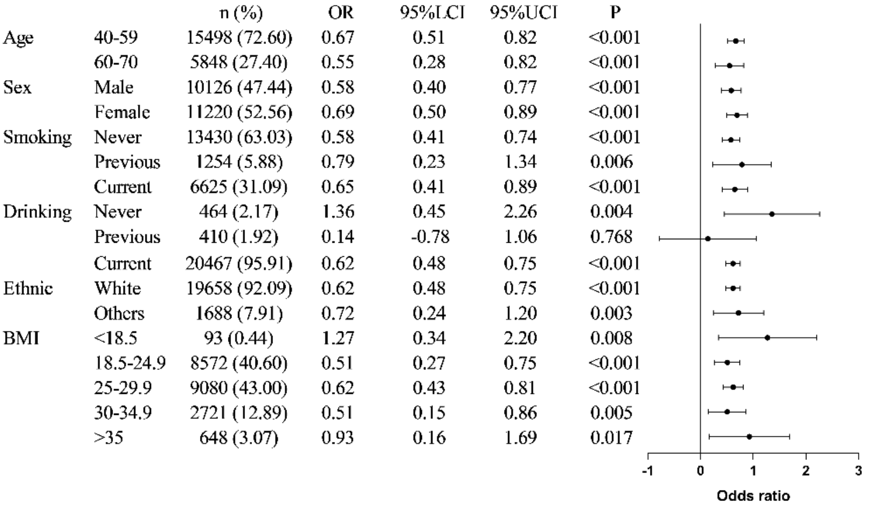

(A)

		n (%)	OR	95%LCI	95%UCI	P
Age	40-60	100 (44.84)	6.29	3.45	9.12	<0.001
	>60	123 (55.16)	4.85	1.23	8.48	0.010
Sex	Male	113 (50.45)	7.24	3.56	10.91	<0.001
	Female	111(49.55)	3.97	0.90	7.04	0.013
Smoking	Yes	53 (23.66)	7.17	1.92	12.43	0.010
	No	171 (76.34)	4.98	2.29	7.68	<0.001
Drinking	Yes	87 (39.19)	6.24	2.36	10.13	0.002
	no	135 (60.81)	4.54	1.44	7.64	0.005
Education	>=16	64 (28.57)	5.95	1.97	9.94	0.005
	<16	160 (71.43)	5.10	2.12	8.07	<0.001

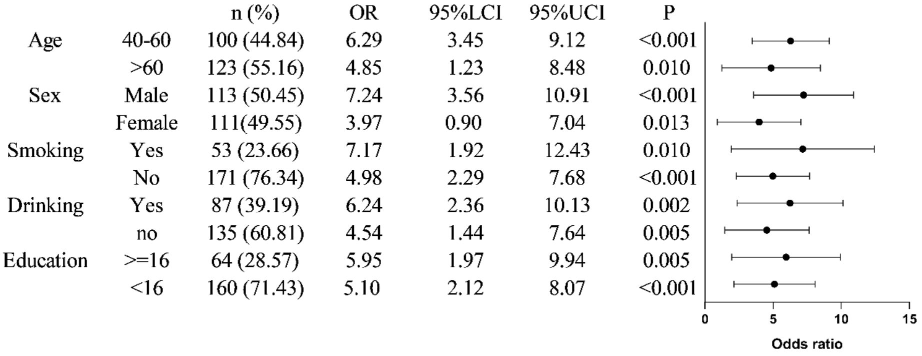

(B)

		n(%)	OR	95%LCI	95%UCI	P
Age	40-60	100 (44.84)	0.19	-0.01	0.40	0.063
	>60	123 (55.16)	0.32	0.09	0.55	0.008
Sex	Male	113 (50.45)	0.32	0.10	0.54	0.006
	Female	111(49.55)	0.10	-0.12	0.33	0.366
Smoking	Yes	53 (23.66)	0.65	0.33	0.96	<0.001
	No	171 (76.34)	0.08	-0.09	0.26	0.354
Drinking	Yes	87 (39.19)	0.49	0.24	0.75	<0.001
	no	135 (60.81)	0.08	-0.09	0.26	0.354
Education	>=16	64 (28.57)	0.02	-0.25	0.30	0.870
	<16	160 (71.43)	0.28	0.09	0.48	0.005

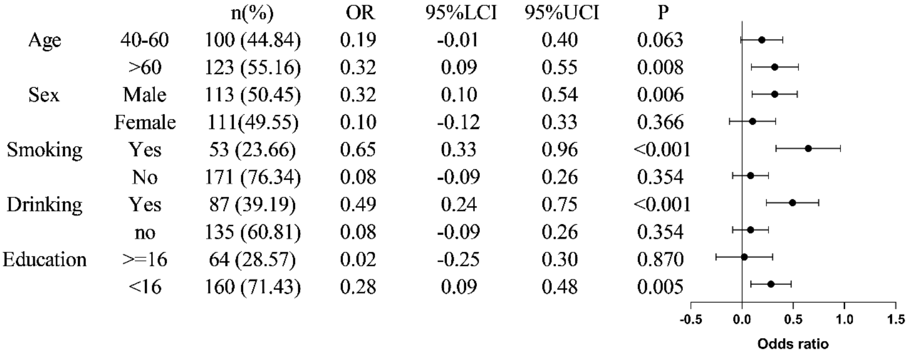

(C)

Figure S4. Subgroup analysis of the association between the mean susceptibility values of striatum and thalamus and metabolic syndrome score in UKB (A). Subgroup analysis of the association between the susceptibility values of right caudate nucleus and metabolic syndrome score (B) and SCWT (C) in Jinan

cohort. Adjusted for age, sex, smoking, drinking status, and education (if not stratified). CI: confidence interval, OR: odds ratio.

Reference

1. Eckstein K, Dymerska B, Bachrata B, et al. Computationally Efficient Combination of Multi-channel Phase Data From Multi-echo Acquisitions (ASPIRE). *Magnetic resonance in medicine*. Jun 2018;79(6):2996-3006. doi:10.1002/mrm.26963
2. Schofield MA, Zhu Y. Fast phase unwrapping algorithm for interferometric applications. *Optics letters*. Jul 15 2003;28(14):1194-6. doi:10.1364/ol.28.001194
3. Wu B, Li W, Avram AV, Gho SM, Liu C. Fast and tissue-optimized mapping of magnetic susceptibility and T2* with multi-echo and multi-shot spirals. *NeuroImage*. Jan 2 2012;59(1):297-305. doi:10.1016/j.neuroimage.2011.07.019
4. Schweser F, Deistung A, Lehr BW, Reichenbach JR. Quantitative imaging of intrinsic magnetic tissue properties using MRI signal phase: an approach to in vivo brain iron metabolism? *NeuroImage*. Feb 14 2011;54(4):2789-807. doi:10.1016/j.neuroimage.2010.10.070
5. Li W, Wang N, Yu F, et al. A method for estimating and removing streaking artifacts in quantitative susceptibility mapping. *NeuroImage*. Mar 2015;108:111-22. doi:10.1016/j.neuroimage.2014.12.043
6. Liu Z, Spincemaille P, Yao Y, Zhang Y, Wang Y. MEDI+0: Morphology enabled dipole inversion with automatic uniform cerebrospinal fluid zero reference for quantitative susceptibility mapping. *Magnetic resonance in medicine*. May 2018;79(5):2795-2803. doi:10.1002/mrm.26946
